# How to estimate R50% for cranial SRS/SRT cases with overlapping 50% isodose volumes: A proposed system

**DOI:** 10.1002/acm2.13624

**Published:** 2022-05-02

**Authors:** Dharmin D. Desai, Ivan L. Cordrey

**Affiliations:** ^1^ Department of Radiation Oncology CHI Memorial Hospital Chattanooga Tennessee USA; ^2^ Regional Cancer Center Cumberland Medical Center Crossville Tennessee USA

**Keywords:** cranial SRS/SRT, fair value estimate, IDC50% overlap, R50%, SIMT

## Abstract

Inevitably in clinical stereotactic cranial single isocenter multiple target cases treated with linac‐based multi‐leaf collimated (MLC) delivery, one encounters planning target volumes (PTVs) in close proximity with overlapping 50% isodose clouds (IDC50%). In such cases, it is very difficult to separate the IDC50% attributable to each individual target and, thus, assess the intermediate dose conformality or R50%. Such scenarios happen regardless of what metric is used to measure intermediate dose spill. Now that universal standards for intermediate dose spill have been proposed, it is important to have a consistent method for apportioning these overlapping IDC50% volumes to allow comparison with the proposed standards when multiple PTVs have overlapping IDC50%. We propose a systematic method for apportioning the IDC50% of multiple targets with overlapping IDC50% based on the relative surface area of each target volume; we call this the fair value estimate (FVE). This FVE system of apportionment is tested for reasonableness by comparing the apportionment of multiple target single isocenter stereotactic treatment with widely spaced targets where the IDC50% can be obviously assigned to demonstrate that the FVE results are very similar to the actual R50% results. We then demonstrate how the FVE system would be applied to cases with overlapping IDC50%. We propose this FVE system for consideration by the cranial stereotactic community for apportioning the intermediate dose spill when that intermediate dose spill overlaps among multiple targets.

## 1 INTRODUCTION

The treatment of single isocenter multiple target (SIMT) stereotactic cranial cases with linac‐based MLC‐collimated delivery is gaining wider implementation in large academic centers and community‐based radiation therapy centers. An important goal in such treatment is ensuring that the intermediate dose spill outside the planning target volumes (PTVs) is minimized. An important metric for intermediate dose spill is R50%, the ratio of the volumes of the 50% isodose cloud (IDC50%) to the PTV volume (*V*_PTV_).

Minimizing normal brain tissue dose is also an important optimization and planning objective in cranial stereotactic treatment because normal brain is an organ at risk (OAR) always directly adjacent to PTV surfaces and subject to the higher doses being delivered to these PTVs. Indeed, radiation necrosis of normal brain tissue is one of the more relevant adverse effects after stereotactic radiosurgery (SRS) and stereotactic radiotherapy (SRT).^1^ Brain radionecrosis has been correlated with the volume of brain that receives a dose of 12 Gy (V12Gy) for single fraction SRS and V18Gy for multi‐fraction SRT.^2^, ^3^ Intermediate dose spill is often tracked and reported in SRS/SRT studies by various metrics. The computation of these metrics often utilizes the volume that receives at least 50% of the prescription dose (Rx), which we refer to as *V*_IDC50%_. Because isodose lines are concentrically nested and SRS/SRT prescriptions are often between 16 and 24 Gy, the *V*_IDC50%_ is often close to V8Gy or V12Gy.^4^ Thus, *V*_IDC50%_ and intermediate dose spill metrics derived from it are reasonable surrogates for normal brain radionecrosis.

Methods for minimizing R50% and appropriate optimization goals for R50% values have been systematically developed.^5^ Applying these R50% minimization methods or any other intermediate dose spill metric depends on being able to determine the R50% of each PTV. This requires a determination of the IDC50% for each PTV, yet inevitably in clinical applications, one encounters clinical presentations of PTVs for which the PTVs are too close together to resolve the individual IDC50%, that is, the IDC50% of closely spaced PTVs overlap. Ballangrud et al.^6^ noted that, “Out of 188 lesions, 13 were so close that the 50% isodose was not split between the two lesions. These lesions were excluded from this GI analysis.”

Further, as mentioned by Popple et al.,^7^ “When targets are sufficiently close together, the 50% and 100% merge, resulting in a large value for the gradient and conformity index. For HyperArc, 10.8% of the targets had bridging at the 50% level….” The large values result from assigning the entire IDC50% for the assembly of closely spaced PTVs to each individual PTV without attempting to apportion the IDC50% volume among the PTVs.

The work of Cui et al.^8^ stated that, “In case that two isodose volumes from two closely located targets are connected, voxels in the connected isodose volume are assigned to a particular target based on the distance from the voxel to the surface of the target. Each voxel in the connected isodose volume is only assigned to one closest target, dividing the connected volume into two sub‐volumes.” This is certainly one system for apportioning the overlapping IDC50% among the involved PTVs, yet this simple proximity‐based division IDC50% could yield very large IDC50% values for small PTVs close to large PTVs.

Now that universal standards for R50% in SRS/SRT have been proposed by Desai et al.,^9^ it is important to be able to compare the R50% of a PTV to those standards to assess plan quality. As stated earlier from the work of Popple et al.,^7^ roughly 11% of SRS/SRT targets likely have overlapping or bridging IDC50%, yet, for these targets, it is impossible to make a comparison to these quality metrics unless the overlapping IDC50% can be separated/apportioned to individual PTVs.

In this work, we explore an apportioning system to account for the overlapping IDC50% of two or more PTVs and to determine the IDC50% of the individual PTVs more appropriately. This accounting system is based on the characteristics of the PTVs with overlapping IDC50%.

## 2 METHODS

### 2.1 Derivation of FVE

When the IDC50% volume is not clearly distinguishable as associated with an individual PTV, as in the case of two PTVs that are close together, we need to systematically distribute the IDC50% volume (*V*_IDC50%_) with a consistent set of rules. Clearly, every PTV has an ICD50% volume that inherently belongs to that PTV. The work of Desai et al.^10^ demonstrates that the minimum value for this IDC50% volume for PTV*_i_* ($V_{\mathrm{IDC}50{\%}_{R50{\%}_{\mathrm{Analytic}}}}^{\mathrm{PT}V_{i}}$) can be extracted from the R50%_Analytic_.

(1) $$V_{\mathrm{IDC}50{\%}_{R50{\%}_{\mathrm{Analytic}}}}^{\mathrm{PT}V_{i}}\;=\;V_{\mathrm{PT}V_{i}}{\;\times \;R50\%}_{\mathrm{Analytic}}^{\mathrm{PT}V_{i}}$$

Thus, there is a minimum *V*_IDC50%_ that is easily attributable to the individual PTVs of the plan based on R50%_Analytic_.

(2) $$V_{\mathrm{IDC}50{\%}_{R50{\%}_{\mathrm{Analytic}}}\;}=\;\sum_{n\;=\;1}^{N}\left(V_{\mathrm{PT}V_{n}}\;\times \;{R50\%}_{\mathrm{Analytic}}^{\mathrm{PT}V_{n}}\right)$$

where *N* is the total number of PTVs with overlapping IDC50%.

This easily attributable portion of the *V*_IDC50%_ is then subtracted from the total *V*_IDC50%_ of the PTVs that share the IDC50% to find the remaining volume of IDC50% that needs to be assigned:

(3) $$V_{\mathrm{IDC}50\%}^{\mathrm{Remaining}}\;=\;V_{\mathrm{IDC}50\%}^{\mathrm{Total}}\;-\;\sum_{n\;=\;1}^{N}\left(V_{\mathrm{PT}V_{n}}{\;\times \;R50\%}_{\mathrm{Analytic}}^{\mathrm{PT}V_{n}}\;\right)$$

When apportioning this $V_{\mathrm{IDC}50\%}^{\mathrm{Remaining}}$, each PTV should be assigned its fair portion based on an objective accounting system that recognizes that different PTV will contribute a different amount to the *V*_IDC50%_ based on the individual characteristics of the PTV. This portion of *V*_IDC50%_ will be referred to as the fair value estimate (FVE). Previous published work studying the “surface area effect” and the derivation of R50%_Analytic_ implies an accounting system based on PTV surface area (SA_PTV_) may prove useful.^10^, ^11^, ^12^ We can assign the $V_{\mathrm{IDC}50\%}^{\mathrm{Remaining}}$ according to the surface area ratio.

$V_{\mathrm{FVE}}^{\mathrm{PT}V_{i}}\;$is that portion of the $V_{\mathrm{IDC}50\%}^{\mathrm{Remaining}}$ assigned to PTV*_i_* and is the surface area‐based FVE.

(4) $$\begin{array}{ccc} & & V_{\mathrm{FVE}}^{\mathrm{PT}V_{i}}=\frac{SA_{\mathrm{PT}V_{i}}}{\sum_{n=1}^{N}SA_{\mathrm{PT}V_{n}}}\;\times \;V_{\mathrm{IDC}50\%}^{\mathrm{Remaining}}\;=\;\frac{SA_{\mathrm{PT}V_{i}}}{\sum_{n\;=\;1}^{N}SA_{\mathrm{PT}V_{n}}}\\ & & \;\;\times \;\left[V_{\mathrm{IDC}50\%}^{\mathrm{Total}}-\;\sum_{n\;=\;1}^{N}\left(V_{\mathrm{PT}V_{n}}{\;\times \;R50\%}_{\mathrm{Analytic}}^{\mathrm{PT}V_{n}}\right)\right] \end{array}$$

The essence of the SA_PTV_‐based accounting system is the surface area ratio (individual PTV surface area/total surface area of all PTVs that share the IDC50%), which determines how much of the “remaining” IDC50% volume gets assigned to each PTV.

The resulting $V_{\mathrm{FVE}}^{\mathrm{PT}V_{i}}$ value is now used to find the complete FVE for R50% for PTV_i_:

(5) $${R50\%}_{\mathrm{FVE}}^{\mathrm{PT}V_{i}}\;=\;\frac{V_{\mathrm{IDC}50{\%}_{R50{\%}_{\mathrm{Analytic}}}}^{\mathrm{PT}V_{i}}\;+\;V_{\mathrm{FVE}}^{\mathrm{PT}V_{i}}\;}{V_{\mathrm{PT}V_{i}}}$$

Using the definition of R50% [Equation (1)] for the first term of Equation (5) and substituting Equation (4) for the second term of Equation (5) gives the following equation for ${R50\%}_{\mathrm{FVE}}^{\mathrm{PT}V_{i}}$:

(6) $${R50\%}_{\mathrm{FVE}}^{\mathrm{PT}V_{i}}\;=\;\frac{V_{\mathrm{PT}V_{i}}{\;\times \;R50\%}_{\mathrm{Analytic}}^{\mathrm{PT}V_{i}}\;+\;\frac{SA_{\mathrm{PT}V_{i}}}{\sum_{n\;=\;1}^{N}SA_{\mathrm{PT}V_{n}}}\;\left[V_{\mathrm{IDC}50\%}^{\mathrm{Total}}\;-\;\sum_{n\;=\;1}^{N}\left(V_{\mathrm{PT}V_{n}}{\;\times \;R50\%}_{\mathrm{Analytic}}^{\mathrm{PT}V_{n}}\right)\right]\;}{V_{\mathrm{PT}V_{i}}}$$

Further simplification of Equation (6) provides the final expression for the FVE for R50% in the PTV surface area‐based accounting system:

(7) $$\begin{array}{ccc} & & {R50\%}_{\mathrm{FVE}}^{\mathrm{PT}V_{i}}{\;=\;R50\%}_{\mathrm{Analytic}}^{\mathrm{PT}V_{i}}\\ & & \;\;+\;\frac{\frac{SA_{\mathrm{PT}V_{i}}}{\sum_{n\;=\;1}^{N}SA_{\mathrm{PT}V_{n}}}\left[V_{\mathrm{IDC}50\%}^{\mathrm{Total}}\;-\;\sum_{n\;=\;1}^{N}\left(V_{\mathrm{PT}V_{n}}{\;\times \;R50\%}_{\mathrm{Analytic}}^{\mathrm{PT}V_{n}}\right)\right]}{V_{\mathrm{PT}V_{i}}} \end{array}$$

As a special case, consider a plan with only one PTV where *i* = 1 and *N* = 1. In such a case, the surface area ratio reduces to unity, and the square brackets reduce to $V_{\mathrm{IDC}50\%}^{\mathrm{Total}}$ ‐ R50%_Analytic_. Thus, the R50%_Analytic_ terms subtract out, and the expression reduces to *V*_IDC50%_/*V*_PTV_, which is exactly the classic definition of R50% for a single PTV plan.

### 2.2 Assessment of FVE

The above accounting system is intended for use when the IDC50% of two or more PTVs overlap (cannot be clearly distinguished). Yet, if the accounting system is to have any validity, it must give reasonable answers even when applied to cases where the IDC50%s of individual PTV are clearly separated (clearly distinguishable). In such cases, the correct answer for the apportioning of the *V*_IDC50%_ is known from the actual planned dose results—the IDC50% can be simply extracted from the treatment planning system (TPS). Answers so extracted from the TPS can be compared directly to the proposed FVE accounting system. To this end, we apply the FVE accounting system to some well‐controlled phantom studies published previously that have clearly distinguishable IDC50% volumes associated with particular PTVs.^5^, ^13^

From Desai et al.,^5^ we have data for three single isocenter multiple target cases with five PTVs of unequal volumes and surface areas. The reader referred to the original published article for the planning details.

Article by Cordrey et al.^13^ yields four single isocenter multiple target cases with six PTVs in each plan. The reader referred to the original published article for the planning details.

From these single isocenter multiple target cases with clearly separated IDC50%, we need the actual R50% (IDC50%) for each PTV (from the TPS) and calculate the R50%_FVE_ for comparison (all needed information is given in the original articles). The FVE accounting system will be applied as if IDC50% of all PTVs overlap, thus the $V_{\mathrm{IDC}50\%}^{\mathrm{Total}}$ of Equation (3) is the IDC50% of the entire plan even though the IDC50% is clearly distinguishable and FVE would not be used clinically in such a circumstance. If the FVE accounting system is reasonable, it must give reasonable answers for each individual PTVs R50% compared to the directly measured R50% (IDC50%) from the TPS.

### 2.3 Application of FVE

To demonstrate the application of R50%_FVE_ accounting, we apply FVE to case studies where, unlike the tests in Section 2.2, the IDC50% cannot be simply assigned to a particular PTV because the PTVs are in close proximity where the IDC50%s overlap.

For this study, we utilize the computed tomography (CT) data set provided by the Radiosurgery Society (RSS), Cases 1 and 2 (The Radiosurgery Society, San Jose, CA, USA). This high‐resolution CT study contains 417 images with a 1 mm slice spacing and 0.9 × 0.9 mm pixel dimensions. The Case 2 data contain a structure set with PTVs and includes OAR contours. OAR structure contours provided in Case 2 include left and right globes of the eye, left and right optic nerves, optic chiasm, pituitary, and brainstem. We retained the OAR structure contours but created study‐specific PTVs as described below.

The treatment plans of this FVE application demonstration were created and optimized in the Eclipse TPS using photon optimizer v15.6 with a final calculation via the AAA v15.6 algorithm on a 1 mm calculation grid size. Plans were created for delivery on a Varian TrueBeam using 6 MV flattening filter‐free photon volumetric modulated arc therapy (VMAT) RapidArc delivery (Varian Medical Systems, Palo Alto, CA, USA).

The beam geometry consists of axial arcs and vertex arcs (with the treatment couch angle at 90° from the 0° position). The optimization used the “ask for it” (AFI) strategy optimization technique with R50%_Goal_ = R50%_Analytic_; a concise description of the AFI strategy is given by Cordrey et al.^13^

In these cases, PTVs in close proximity that are of unequal volume and surface area are created. As simple demonstrations of FVE accounting, we examine a two PTV case and a three PTV case. The IDC50% information is extracted along with the PTV characteristics. The IDC50% volumes overlap and cannot be visually separated, so the R50%_FVE_ [Equation (7)] is computed and presented for consideration. The details of the calculations for the application of the FVE accounting system to the two PTV are given in Appendix A.

Finally, the FVE accounting system is applied to a complex nine PTV single isocenter multiple target case. The location and size of the PTVs are inspired by a clinical case in which there is one very large target and eight much smaller targets. This case is modeled in the RSS Case 1 phantom and planned as described above. Four small PTVs have clearly distinct IDC50% volumes for which the FVE accounting system is not needed and, thus, will not be used. There are two clusters of PTVs for which the *V*_IDC50%_ of two or more PTVs overlap and cannot be easily separated. One such overlapping IDC50% volume involved a very large PTV (*V*_PTV_ = 21.12 cm^3^) and two much smaller PTVs (*V*_PTV_ = 0.72 and 0.20 cm^3^) in the anterior of the cranium. The other cluster of overlapping IDC50% is in the posterior of the cranium and involves two PTVs of volumes 0.34 and 0.87 cm^3^. The FVE accounting system will be applied to the overlapping IDC50% in the anterior of the cranium and separately to the overlapping IDC50% in the posterior of the cranium.

## 3 RESULTS

### 3.1 Assessment of FVE

**FIGURE 1:**
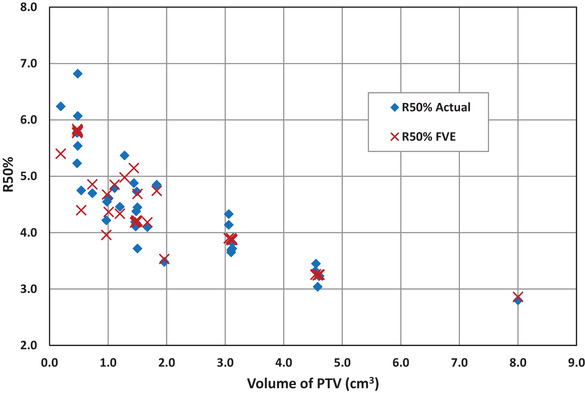
Comparison plot of R50%_Actual_ and R50%_FVE_ versus planning target volume (PTV) volume for the cases listed in Table 1. There are six R50%_FVE_ values at nearly the same *V*_PTV_ at nominal values of 0.48, 1.48, 3.1, and 4.58 cm^3^. Because there are six data points at these volumes, the data markers are nearly coincident and can easily be mistaken for a bold “X.” Notice the R50%_Actual_ and R50%_FVE_ values fall in the same general band, which indicates that the fair value estimate (FVE) accounting system is a reasonable approximation of the R50%_Actual_

The basic planning results are summarized in the original articles from which these data are extracted. The relevant data for application of the R50% FVE are summarized in Table 1, along with the comparison of the actual R50% for each PTV. In these cases, we know the actual R50% for each PTV because these are well‐separated PTVs with clearly distinguishable IDC50% volumes. The difference between R50%_FVE_ and the actual R50% is listed in Table 1. R50%_FVE_ and R50%_Actual_ are plotted as a function of PTV volume in Figure 1. The mean difference between R50%_Actual_ and R50%_FVE_ is ‐0.05 ± 0.31.

**TABLE 1:** Assessing the R50% fair value estimate (FVE) accounting system

	PTV #	Vol (cm**^3^**)	SA (cm**^2^**)	R50%_Analytic_	$V_{\mathrm{IDC}50{\%}_{R50{\%}_{\mathrm{Analytic}}}}$ (cm**^3^**)	*V*_FVE_ (cm**^3^**)	R50%_FVE_	R50%_Actual_	Difference	$V_{\mathrm{IDC}50\%}^{\mathrm{Total}}$ (cm**^3^**)	$V_{\mathrm{IDC}50\%}^{\mathrm{Remaining}}$ (cm**^3^**)
Plan 1										37.05	6.53
	1	0.54	3.17	3.34	1.80	0.57	4.40	4.75	−0.35		
	2	1.96	7.52	2.84	5.57	1.36	3.53	3.48	0.05		
	3	0.19	1.59	3.89	0.74	0.29	5.40	6.24	−0.84		
	4	0.97	4.68	3.09	3.00	0.85	3.96	4.22	−0.26		
	5	8.00	19.22	2.43	19.41	3.47	2.86	2.80	0.06		
Plan 2										24.73	6.13
	1	1.20	6.11	3.27	3.93	1.28	4.34	4.46	−0.12		
	2	0.98	5.61	3.48	3.41	1.17	4.68	4.55	0.13		
	3	0.73	4.51	3.57	2.60	0.94	4.86	4.70	0.16		
	4	1.01	5.28	3.28	3.31	1.10	4.37	4.61	−0.24		
	5	1.67	7.85	3.20	5.35	1.64	4.18	4.10	0.08		
Plan 3										34.88	9.37
	1	1.50	7.96	3.45	5.17	1.86	4.69	4.45	0.24		
	2	1.44	8.62	3.74	5.39	2.02	5.15	4.88	0.27		
	3	1.11	6.33	3.52	3.90	1.48	4.85	4.79	0.06		
	4	1.83	9.66	3.51	6.42	2.26	4.74	4.85	−0.11		
	5	1.28	7.44	3.62	4.63	1.74	4.98	5.37	−0.39		
Plan 4										16.52	5.71
	1	0.48	3.44	3.81	1.83	0.96	5.82	5.54	0.28		
	2	0.47	3.39	3.82	1.79	0.95	5.84	5.77	0.07		
	3	0.47	3.34	3.78	1.77	0.94	5.77	5.23	0.54		
	4	0.47	3.34	3.78	1.77	0.94	5.77	5.78	−0.01		
	5	0.48	3.43	3.80	1.82	0.96	5.80	6.07	−0.27		
	6	0.48	3.42	3.79	1.82	0.96	5.79	6.82	−1.03		
Plan 5										37.27	9.45
	1	1.50	6.97	3.14	4.71	1.60	4.21	3.72	0.49		
	2	1.48	6.85	3.13	4.63	1.57	4.19	4.17	0.02		
	3	1.46	6.74	3.12	4.56	1.55	4.18	4.19	−0.01		
	4	1.47	6.81	3.13	4.60	1.56	4.19	4.11	0.08		
	5	1.48	6.90	3.15	4.65	1.58	4.22	4.38	−0.16		
	6	1.49	6.89	3.13	4.66	1.58	4.19	4.73	−0.54		
Plan 6										72.15	20.09
	1	3.10	10.95	2.81	8.72	3.37	3.90	3.65	0.25		
	2	3.10	10.84	2.80	8.67	3.34	3.87	3.69	0.18		
	3	3.12	10.89	2.79	8.72	3.35	3.87	3.72	0.15		
	4	3.12	10.94	2.80	8.74	3.37	3.88	3.82	0.06		
	5	3.06	10.82	2.81	8.61	3.33	3.90	4.14	−0.24		
	6	3.06	10.82	2.81	8.61	3.33	3.90	4.33	−0.43		
Plan 7										89.45	16.08
	1	4.60	14.12	2.67	12.30	2.70	3.26	3.24	0.02		
	2	4.58	13.96	2.66	12.19	2.67	3.24	3.04	0.20		
	3	4.61	14.12	2.67	12.31	2.70	3.26	3.23	0.03		
	4	4.60	14.09	2.67	12.28	2.69	3.25	3.23	0.02		
	5	4.54	13.93	2.67	12.12	2.66	3.26	3.32	−0.06		
	6	4.55	14.00	2.67	12.17	2.67	3.26	3.45	−0.19		

*Note*: The data given are summary data from previously published work (through private communication),^1^, ^7^ and each plan is a single isocenter multiple target case for well‐separated planning target volumes (PTVs). The 50% isodose clouds (IDC50%) for each PTV is clearly encompassing only one PTV each, and as such, the IDC50% associated with each PTV is unambiguously identified. Thus, R50%_Actual_ is considered “the correct answer.” *V*_PTV_ is obtained directly from the treatment planning system. SA_PTV_ is obtained from a surface area script in the treatment planning system.^6^ R50%_Analytic_ is the semi‐empirical minimum R50% value^5^ from which the $V_{\mathrm{IDC}50{\%}_{R50{\%}_{\mathrm{Analytic}}}}$is derived [Equation (1)]. *V*_FVE_ is the volume of additional (more than R50%_Analytic_) IDC50% assigned to a PTV as calculated by Equation (4). R50%_FVE_ is the final result for the FVE for R50% given by Equation (7). In these cases, the FVE accounting system is not truly needed because the IDC50% of each PTV is easily identified, but this does provide a method for assessing the validity of the FVE in cases when the true answer is known from the planning study (R50%_Actual_). The difference listed is R50%_FVE_ − R50%_Actual_.

### 3.2 Application of FVE

**FIGURE 2:**
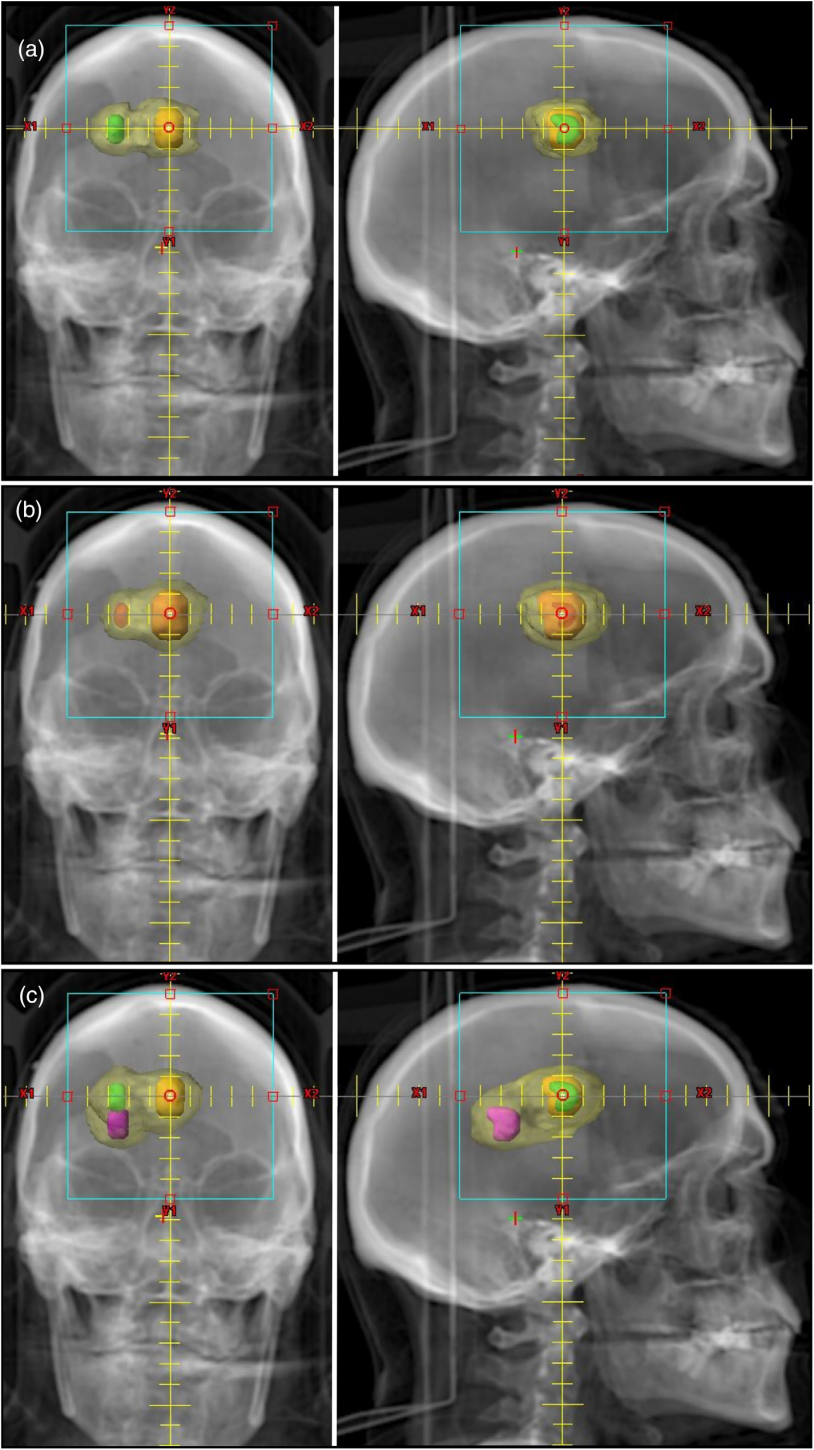
DRR images of the Radiosurgery Society (RSS) Case 2 phantom illustrating planning target volumes (PTVs) that have overlapping 50% isodose clouds (IDC50%) volumes. The solid color shapes are the PTVs. The transparent yellow volume is the overlapping IDC50% that must be separated by the fair value estimate (FVE) accounting system. (a) The AP and lateral DRRs of a two PTVs of different volumes and surface areas and their associated overlapping IDC50% volume. (b) The AP and lateral DRRs of two PTVs with a larger volume and surface area difference. (c) The AP and lateral DRRs of a three PTV case. The quantitative data for these cases are given in Table 2

Figure 2 displays the PTVs and the associated IDC50% for an anterior‐posterior (AP) and lateral digitally reconstructed radiographs (DRR) for cases where the IDC50% volumes of nearby PTVs overlap (for a two PTV case and a three PTV case). The IDC50% for these closely spaced PTVs cannot be visually separated, and thus there is no “actual” R50% value available for comparison. The values for R50%_FVE_ are given in Table 2 for these cases.

**TABLE 2:** Applying the R50% fair value estimate (FVE) accounting system

	PTV #	Vol (cm^3^)	SA (cm^2^)	R50%_Analytic_	$V_{\mathrm{IDC}50{\%}_{R50{\%}_{\mathrm{Analytic}}}}$ (cm^3^)	*V*_FVE_ (cm^3^)	R50%_FVE_	$V_{\mathrm{IDC}50\%}^{\mathrm{Total}}$ (cm^3^)	$V_{\mathrm{IDC}50\%}^{\mathrm{Remaining}}$ (cm^3^)
2PTVs‐1Iso								29.34	15.60
	1	1.02	5.46	3.33	3.40	4.76	8.00		
	2	3.76	12.44	2.75	10.34	10.84	5.63		
2PTVsLrgSml								40.40	21.03
	1	0.47	3.41	3.83	1.80	3.29	10.84		
	2	6.91	18.36	2.54	17.57	17.74	5.11		
3PTVs‐1Iso								57.34	38.28
	1	1.02	5.46	3.33	3.40	8.20	11.37		
	2	3.76	12.44	2.75	10.34	18.69	7.72		
	3	1.75	7.58	3.04	5.32	11.39	9.55		

*Note*: Each plan is a single isocenter multiple target case for planning target volumes (PTVs) in close proximity such that the 50% isodose clouds (IDC50%) of the individual PTVs overlap. *V*_IDC50%_ cannot be easily assigned to the individual PTVs without some objective accounting system for the IDC50%, and as a result, individual R50% values cannot be determined for the PTVs. R50%_Analytic_ is the semi‐empirical minimum R50% value^5^ from which the $V_{\mathrm{IDC}50{\%}_{R50{\%}_{\mathrm{Analytic}}}}$is derived [Equation (1)]. *V*_FVE_ is the volume of additional IDC50% assigned to a PTV as calculated by Equation (4). R50%_FVE_ is the final result for the FVE for R50% given by Equation (7). For these cases, there is no “actual” value of R50% for comparison because there is no unambiguous method or universally accepted accounting system for assigning the overlapping IDC50%.

**FIGURE 3:**
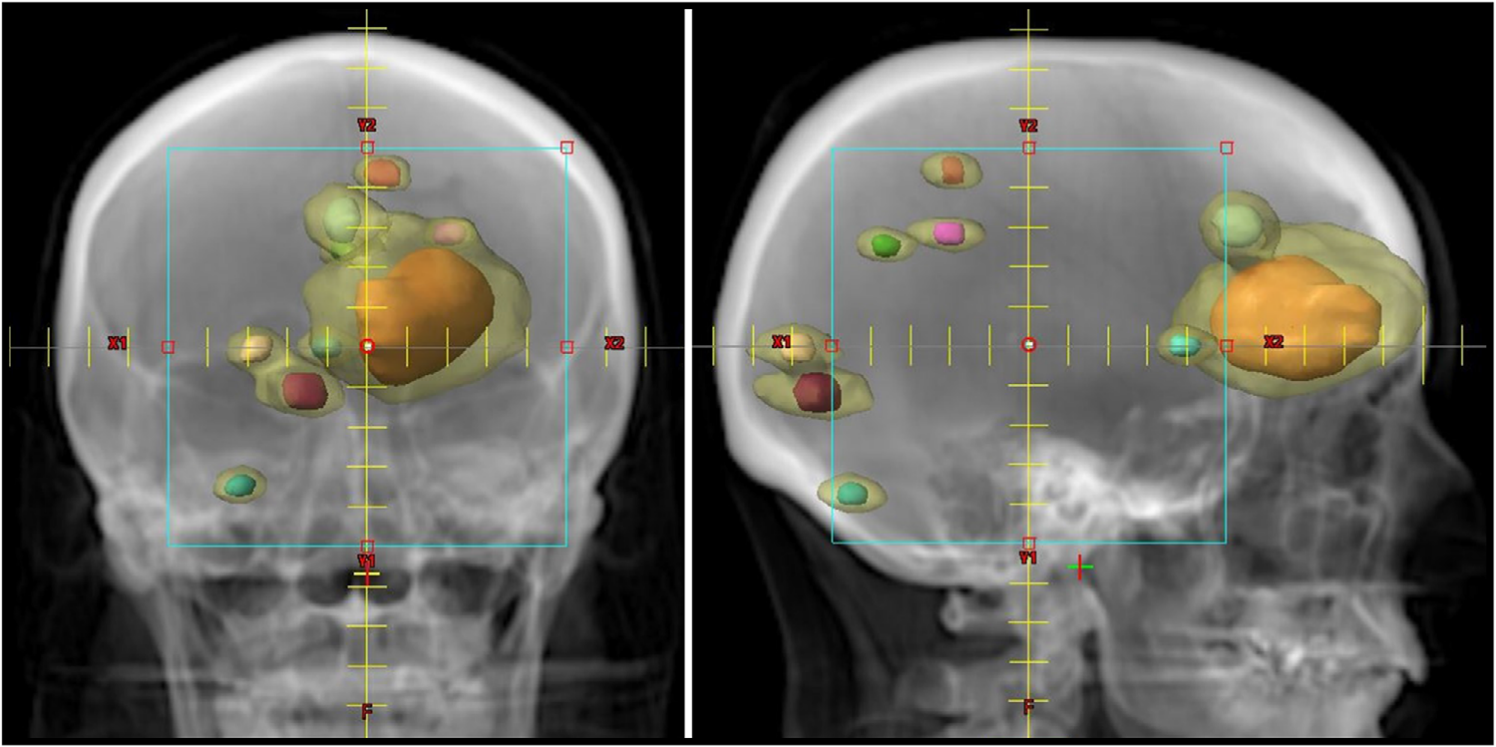
DRR images of the Radiosurgery Society (RSS) Case 1 phantom illustrating the nine planning target volume (PTV) case. There are two clusters of PTVs that have overlapping 50% isodose clouds (IDC50%) volumes for which we apply the fair value estimate (FVE) accounting system. The solid color shapes are the PTVs. The transparent yellow volume is the IDC50%. There are two overlapping IDC50% regions that must be separated by the FVE accounting system: (1) the three PTVs in the anterior cranium clustered around the large orange PTV and (2) the two PTVs in the posterior cranium clustered around the brown PTV. The quantitative data for this example are given in Table 3

Figure 3 displays the PTVs and the associated IDC50% for an AP and lateral DRR for the case with nine PTVs including two clusters of PTVs for which the IDC50% of individual PTVs cannot be visually separated, and as stated above, there is no “actual” R50% value available for comparison. Table 3 lists the data for the application of the FVE for this nine PTV case.

**TABLE 3:** Summary data of fair value estimate (FVE) applied to a nine planning target volume (PTV) single isocenter multiple target case shown in Figure 3

PTV #	Vol (cm^3^)	SA (cm^2^)	R50%_Analytic_	$V_{\mathrm{IDC}50{\%}_{R50{\%}_{\mathrm{Analytic}}}}$ (cm^3^)	*V*_FVE_ (cm^3^)	R50%_FVE_	R50%_Actual_	$V_{\mathrm{IDC}50\%}^{\mathrm{Total}}$ (cm^3^)	$V_{\mathrm{IDC}50\%}^{\mathrm{Remaining}}$ (cm^3^)
								4.76	1.20
1	0.25	2.08	–	–	–	–	5.16		
4	0.28	2.15	–	–	–	–	4.82		
6	0.17	1.57	–	–	–	–	5.76		
9	0.20	1.71	–	–	–	–	5.70		
								67.49	17.64
2	0.72	4.08	3.35	2.41	1.62	5.60	–		
3	20.12	38.61	2.32	46.64	15.33	3.08	–		
5	0.20	1.74	4.02	0.80	0.69	7.48	–		
								6.75	2.66
7	0.34	2.46	3.70	1.26	0.93	6.43	–		
8	0.87	4.60	3.25	2.83	1.73	5.25	–		

*Note*: The first four PTVs (1, 4, 6, 9) have clearly distinguishable 50% isodose clouds (ICD50%) volumes, and FVE is not used for them. The second group of three PTVs (2, 3, 5) are a cluster with overlapping IDC50% for which FVE is used to assign the IDC50% volume ($V_{\mathrm{IDC}50\%}^{\mathrm{Total}}$) within that cluster. $V_{\mathrm{IDC}50\%}^{\mathrm{Remaining}}$ is the volume of IDC50% that cannot be assigned to a PTV based on R50%_Analytic_ and is assigned based on Equation (4), which allows for the calculation of R50%_FVE_ from Equation (7). Similarly, the last two PTVs (7, 8) are a cluster of PTVs with overlapping IDC50% for which FVE is applied separately within this cluster of two PTVs.

## 4 DISCUSSION

The results presented in Table 1 and Figure 1 compare the FVE R50% (R50%_FVE_) with the actual R50% in the cases with clearly distinct IDC50% volumes for each PTV in the single isocenter multiple target plans. Because the *V*_IDC50%_ is clearly separated into volumes each associated with a specific PTV, the FVE accounting system is not needed; however, this comparison does give insight regarding the reasonableness of the accounting system. Here, the actual answer is known, and thus, we have something to compare against to test the FVE accounting system. Figure 1 provides a graphical comparison of the difference between the R50%_Actual_ and the R50%_FVE_. This figure shows that the R50%_FVE_ is a reasonable approximation of the R50%_Actual_ as can be seen in the way that the R50% values cluster in the same band of values. One also sees that the difference values (R50%_FVE_ − R50%_Actual_) given in Table 1 do not vary widely from zero. The mean difference value of R50%_FVE_ − R50%_Actual_ is −0.05 ± 0.31. Thus, there is a remarkably small difference between the R50%_Actual_ and the estimated R50%_FVE_ for this *V*_PTV_ range (0.19–8.0 cm^3^).

A plan that demonstrates that FVE provides very reasonable R50%_FVE_ values for large differences in *V*_PTV_ is Plan 1 (Table 1). This is a five PTV plan which includes a *V*_PTV_ = 8.0 and 0.19 cm^3^ with R50% difference of 0.06 and ‐0.84, respectively. It is not surprising that the large PTV has a small R50% difference because of the *V*_PTV_ normalization of R50%; a small variance in *V*_IDC50%_ becomes even smaller when divided by a large *V*_PTV_. Yet, the small volume PTV's R50%_FVE_ variance from the R50%_Actual_ of −0.84 tells us the FVE predicted *V*_IDC50%_ is only a 0.16 cm^3^ difference in *V*_IDC50%_, which is of debatable clinical relevance and is often within the statistical noise of the TPS extraction of the volume of IDC50%.

From this assessment of R50%_FVE_, we conclude the FVE accounting system based on the PTV surface area is a reasonable approximation for the actual clinically realized answer for R50%. Further, as was shown in Section 2.1, for a single PTV plan the FVE formalisms reduces to the obvious correct answer. Thus, FVE accounting system appears reasonable in specialized cases when the true answer is known.

Having established reasonableness for the FVE accounting system in cases where the answer is already known, we apply the FVE to cases in which the R50% cannot be known by simple observation of the IDC50%—cases when the ICD50% overlaps and cannot be visually separated. We present a two PTV case and a three PTV case in Table 2. By design, the FVE accounting system accounts for the entire IDC50% volume. Clearly, the FVE accounting system works as easily for three PTVs as for two PTVs and would be easy to apply to four or more PTVs if needed. There is currently no obvious way to evaluate this partitioning of the IDC50%, which is precisely the point of this FVE accounting system. We are proposing the FVE accounting system for use in evaluating the R50% of PTVs in clinical scenarios where the IDC50% is not clearly separated and associated with a specific PTV but rather the IDC50% is a merged/overlapping volume that encompasses multiple PTVs. FVE is one possible unambiguous, consistent method for assigning the volume of IDC50%.

Figure 3 and Table 3 present a complex case inspired by a clinical treatment plan. Here we see a nine PTV single isocenter multiple target case in which four PTVs have easily distinguished IDC50% volumes, but the other five PTVs are part of overlapping IDC50% volumes. This is exactly the scenario for which the FVE accounting system was designed. In such a case, the clearly distinguished IDC50% volumes are used directly to calculate R50% in the standard obvious manner. The FVE is applied separately to the two clusters of merged IDC50%. We apply the FVE to the anterior cluster of three PTVs with overlapping IDC50%, and separately apply FVE to the posterior cluster of two PTVs with overlapping IDC50%. This allows us to report an individual R50% for each of the nine PTVs within this complex plan.

With the recent publication of proposed universal quality guidelines for intermediate dose spill measured with R50%,^9^ the FVE accounting system allows comparison with those guidelines even when the individual IDC50% for each PTV cannot be obviously determined. Comparison with these metrics is impossible without a way to apportion the overlapping IDC50% of multiple PTV. In addition, the FVE system may be modified to be applicable if a metric for intermediate dose spill other than R50% is chosen.

Clinical data would not reveal more than the phantom studies because, just like the phantom cases, we cannot clearly separate the IDC50% of an individual PTV from the merged IDC50% that envelopes the close PTVs. Further, it is not necessary to have a well‐optimized plan with small IDC50% to apply the FVE accounting system. The results of the FVE are probably more reliable for well‐optimized plans because the $V_{\mathrm{IDC}50\%}^{\mathrm{Remaining}}$ that must be apportioned is smaller, but it is impossible to fully assess this because, by its nature, the IDC50% for an individual PTV in a collection of PTVs with overlapping IDC50%s cannot be obviously known; but it can be unambiguously determined by the FVE accounting system.

An interesting aspect of the surface area weighting [shown mathematically in Equation (4)] is that for a collection of roughly spherical PTVs, the larger the PTV the larger the FVE apportioned IDC50%, that is, for a given PTV shape, as the volume increases so does the surface area: the volume, and surface area of any three‐dimensional object of a specific shape are linked by a constant mathematical relationship for that specific shape. Thus, this surface area weighted apportioning is also a pseudo‐volumetric apportioning that also accounts for the known surface area relationship of R50%.^11^ In the three example demonstrations of the FVE system application (Tables 2 and 3), one sees the larger PTV is apportioned a larger IDC50%.

One advantage of this FVE accounting system over a purely geometric accounting system (one based on pixel proximity to a PTV to assign the IDC50% apportionment) is the ease of use. One does not need a script to assess each pixel, calculate a displacement vector to each PTV, and assign the pixel to a PTV. The FVE accounting system is a simple calculation that can be performed in a spreadsheet with data obtained from the TPS. FVE accounting might even be incorporated into the TPS as a script or into a secondary reporting system like ClearCheck (Radformation, New York, NY, USA). The only challenging piece of data is the PTV surface area which is currently not reported by most TPS. We used a previously validated surface area script to extract that value.^11^ We anticipate that, as the utility of PTV surface area becomes recognized in radiation oncology, surface area will become a standard reported metric by the planning system vendors much like volume is reported currently.^10^, ^11^

If surface area values are not easily accessible (the TPS vendor does not supply them or a surface area script is unavailable), one could approximate the best case FVE by assuming all PTVs are spherical and calculating the surface area ratio and R50%_Analytic_ based on that spherical PTV approximation. This approximation would be reasonable as long as the PTVs are largely spherical, which is true for many SRS/SRT PTVs.

The FVE for R50% is only possible because we have the theoretical framework of R50%_Analytic_ and the surface area effect in which to understand and propose a solution to the issue of overlapping IDC50% volumes for closely spaced PTVs.^10^, ^11^ This is not the only possible accounting system for dealing with overlapping IDC50%.

## 5 CONCLUSION

We propose an accounting system for intermediate dose spill in cranial stereotactic treatment for the roughly 11% of clinical single isocenter multiple target cases with PTVs having merged/overlapping IDC50% volumes. In such cases, a standardized method is needed to assign the IDC50% to the individual PTVs to allow comparison with recently proposed universal quality standards for intermediate dose spill.^9^ When the IDC50% of multiple PTVs overlap, the FVE accounting system for R50% uses the ratio of the individual PTV surface area to the total surface area of all PTVs that share the IDC50% to apportion the IDC50% according to Equation (7). We encourage debate about the merits of this system and any other proposed accounting system that may lead to an accepted standard R50% or IDC50% accounting system when IDC50% overlap occurs for multiple PTVs.

## CONFLICT OF INTEREST

No conflicts of interest.

## FUNDING STATEMENT

There are no funders to report for this submission.

## AUTHOR CONTRIBUTIONS

All authors contributed equally to this project.

## Data Availability

The data that support the findings of this study are available from the corresponding author upon reasonable request.

## APPENDIX A FVE ACCOUNTING SYSTEM DEMONSTRATION

### A.1

A simple example of the FVE accounting system as applied to a two PTV plan is provided below using the data listed for the first plan (2PTVs‐1Iso) in Table 2.

**Step 1**: Extract information directly available from the TPS needed for FVE calculations—*V*_PTV_ and SA_PTV_ for each individual PTV and total *V*_IDC50%_ surrounding the PTVs. *V*_PTV_ was taken directly from the Eclipse TPS, a specialized script was used to extract $SA_{\mathrm{PT}V_{i}}$ values,^10^ and $V_{\mathrm{IDC}50\%}^{\mathrm{Total}}$ was obtained by converting the IDC50% to a structure and reading the volume from the Eclipse TPS.

Example values:

$$V_{\mathrm{PT}V_{1}}=1.02\;cm^{3}$$

$$V_{\mathrm{PT}V_{2}}=3.76\;cm^{3}$$

$$SA_{\mathrm{PT}V_{1}}=\;5.46\;cm^{2}$$

$$SA_{\mathrm{PT}V_{2}}=12.44\;cm^{2}$$

$$V_{\mathrm{IDC}50\%}^{\mathrm{Total}}=29.34\;cm^{3}$$

**Step 2**: Calculate Δ*r*_PTV_, *r*_PTV_, and R50%_Analytic_ for each individual PTV by substituting the values from Step 1 into the following equations:^10^
  (A.1)
  $$\Delta r_{\mathrm{PT}V_{i}}=\left(0.2844\right)\times {\left(V_{\mathrm{PT}V_{i}}\right)}^{0.1973}$$
  (A.2)
  $$r_{\mathrm{PT}V_{i}}=\sqrt[{3}]{\frac{3V_{\mathrm{PT}V_{i}}}{4\pi }}\;$$
  (A.3)
  $$\begin{array}{ccc} & & {R50\%}_{\mathrm{Analytic}}^{\mathrm{PT}V_{i}}=\;1+\;\frac{SA_{\mathrm{PT}V_{i}}}{V_{\mathrm{PT}V_{i}}}\Delta r_{\mathrm{PT}V_{i}}\\ & & \;\left[1+\frac{\Delta r_{\mathrm{PT}V_{i}}}{r_{\mathrm{PT}V_{i}}}+\frac{1}{3}{\left(\frac{\Delta r_{\mathrm{PT}V_{i}}}{r_{\mathrm{PT}V_{i}}}\right)}^{2}\right] \end{array}$$

Example calculations and values:

$$\Delta r_{\mathrm{PT}V_{1\;}}=\;\left(0.2844\right)\;\times \;{\left(1.02\right)}^{0.1973}\;=\;0.29\;\mathrm{cm}$$

$$\Delta r_{\mathrm{PT}V_{2}\;}=\;\left(0.2844\right)\;\times \;{\left(3.76\right)}^{0.1973}\;=\;0.37\;\mathrm{cm}$$

$$r_{\mathrm{PT}V_{1}\;}\;=\;\sqrt[{3}]{\frac{3(1.02)}{4\pi }}\;=\;0.62\;\mathrm{cm}$$

$$r_{\mathrm{PT}V_{2}}\;=\;\sqrt[{3}]{\frac{3(3.76)}{4\pi }}\;=\;0.96\;\mathrm{cm}$$

$$\begin{array}{ccc} & & {R50\%}_{\mathrm{Analytic}\;}^{\mathrm{PT}V_{1}}\\ & & \;\;=\;1\;+\;\frac{5.46}{1.02}0.29\left[1+\frac{0.29}{0.62}+\frac{1}{3}{\left(\frac{0.29}{0.62}\right)}^{2}\right]\;=\;3.33\; \end{array}$$

$$\begin{array}{ccc} & & {R50\%}_{\mathrm{Analytic}\;}^{\mathrm{PT}V_{2}}\\ & & \;\;=\;1\;+\;\frac{12.44}{3.76}0.37\left[1+\frac{0.37}{0.96}+\frac{1}{3}{\left(\frac{0.37}{0.96}\right)}^{2}\right]\;=\;2.75 \end{array}$$

**Step 3**: Calculate R50%_FVE_ for each individual PTV by substituting the appropriate values from Steps 1 and 2 into the following equation [Equation (7) from Section 2]:
  (A.4)
  $$\begin{array}{ccc} & & {R50\%}_{\mathrm{FVE}}^{\mathrm{PT}V_{i}}{\;=\;R50\%}_{\mathrm{Analytic}}^{\mathrm{PT}V_{i}}\\ & & \;+\;\frac{\;\frac{SA_{\mathrm{PT}V_{i}}}{\sum_{n\;=\;1}^{N}SA_{\mathrm{PT}V_{n}}}\;\left[V_{\mathrm{IDC}50\%}^{\mathrm{Total}}\;-\;\sum_{n\;=\;1}^{N}\;\left(V_{\mathrm{PT}V_{n}}{\;\times \;R50\%}_{\mathrm{Analytic}}^{\mathrm{PT}V_{n}}\right)\;\right]\;}{V_{\mathrm{PT}V_{i}}} \end{array}$$

Example calculations and values:

$$\begin{array}{ccc} & & {R50\%}_{\mathrm{FVE}}^{\mathrm{PT}V_{1}}=\;3.33\;\\ & & \;+\frac{\frac{5.46}{(5.46\;+\;12.44)}\left[29.34\;-\;\left(\left(1.02\;\times \;3.33\right)\;+\;\left(3.76\;\times \;2.75\right)\right)\right]}{1.02}=8.00 \end{array}$$

$$\begin{array}{ccc} & & {R50\%}_{\mathrm{FVE}}^{\mathrm{PT}V_{2}}\;=\;2.74\;\\ & & \;+\frac{\frac{12.44}{(5.46\;+\;12.44)}\left[29.34\;-\;\left(\left(1.02\;\times \;3.33\right)\;+\;\left(3.76\;\times \;2.75\right)\right)\right]}{3.76}=5.63 \end{array}$$
